# Neuropathological findings in entorhinal cortex of subjects aged 50 years or older and their correlation with dementia in a sample from Southern Brazil

**DOI:** 10.1590/1980-57642016dn11-010005

**Published:** 2017

**Authors:** Edson Rodrigues Neto, Mariana K. Fonseca, Álvaro C.B. Guedes, Francine H. Oliveira, Arlete Hilbig, Liana Lisboa Fernandez

**Affiliations:** 1 Medical Student at the Federal University of Health Sciences of Porto Alegre (UFCSPA). Scientific initiation scholars of the Foundation for Research of Rio Grande do Sul.; 2 Pathologist. Specialist in Neuropathology at the Institute of Neuropathology of the University Hospital of Zurich. MSc in Pathology at the Federal University of Health Sciences of Porto Alegre, Porto Alegre RS, Brazil.; 3 Neurologist. PhD in Internal Medicine at the Federal University of Rio Grande do Sul, Porto Alegre RS, Brazil. Associate Professor at the Federal University of Health Sciences of Porto Alegre, Porto Alegre RS, Brazil.; 4 Neurologist. PhD in Cellular and Molecular Biology at the Pontifical Catholic University of Rio Grande do Sul, Porto Alegre RS, Brazil and the University of Barcelona, and Adjunct Professor at the Federal University of Health Sciences of Porto Alegre, Porto Alegre RS, Brazil.

**Keywords:** dementia, Alzheimer, entorhinal cortex, immunohistochemistry, anomalous protein deposits, IQCODE

## Abstract

**Introduction:**

The aims of this study were to survey neurodegenerative changes detected by abnormal protein deposits in the Entorhinal Cortex (EC) of subjects aged 50 years or older and to correlate these findings with suspected dementia, as detected by the IQCODE (Informant Questionnaire on Cognitive Decline in the Elderly).

**Methods:**

Fourteen brains were submitted to the immunohistochemistry technique for different proteins (beta-amyloid, tau, α-synuclein and phospho-TDP-43) and data obtained compared with IQCODE scores.

**Results:**

Fifty-seven percent of the individuals exhibited IQCODE results compatible with dementia, being classified into the demented group (DG): 87.5% of patients had neuropathological findings corresponding to Alzheimer's-like brain pathology (ALBP). Of the patients in the non-demented group (NDG), 16.7% met neuropathological criteria for ALBP. All individuals in the DG showed deposits of more than one kind of protein in the EC. The most common association was hyperphosphorylated tau and beta-amyloid protein (87.5%).

**Discussion:**

Most individuals with dementia had neuropathological findings of ALBP, as did one individual with no signs of dementia, characterizing a preclinical stage. The results of this study suggest that deposits of a single type of anomalous protein are normal findings in an aging brain, while more than one kind of protein or the combined presence of anomalous protein deposits indicate the presence of dementia.

## Abstract (pt)

**Introdução:**

Este trabalho visa avaliar alterações neurodegenerativas detectadas por depósitos proteicos anormais em Córtex Entorrinal (CE) de indivíduos acima de 50 anos e correlacionar os achados com suspeição de demência detectada por meio do IQCODE (*Informant Questionnaire on Cognitive Decline in the Elderly*).

**Métodos:**

Catorze encéfalos foram submetidos à técnica imuno-histoquímica para diferentes proteínas (beta-amiloide, tau, alfa-sinucleína e fosfo-TDP-43) e esses dados foram comparados com os valores obtidos pelo IQCODE.

**Resultados:**

57% dos indivíduos mostraram resultados de IQCODE compatíveis com demência, sendo classificados no grupo com demência (GD): 87,5% desses pacientes tinham achados neuropatológicos correspondentes a patologia cerebral Alzheimer-símile (ALBP). Entre os pacientes do grupo sem demência (GSD), 16,7% apresentaram critérios neuropatológicos para ALBP. Todos os indivíduos do GD tinham depósitos de mais de um tipo de proteína no CE. A associação proteica mais comum foi tau hiperfosforilada e proteína beta-amiloide (87,5%).

**Discussão:**

A maioria dos indivíduos com demência apresentaram achados neuropatológicos de ALBP e um indivíduo, que não tinha evidências de demência, apresentou achados compatíveis com ALBP, caracterizando um estágio pré-clínico. Este trabalho sugere que depósitos de um único tipo de proteína anômala são achados normais do cérebro em envelhecimento, enquanto mais de um tipo de proteínas ou a presença combinada de depósitos proteicos anômalos indica manifestações de demência.

## INTRODUCTION

Brazil stands out as a country with high growth rates of the elderly population, with ageing projections of about 32 million people aged over 65 years by 2025. An older population emerges as a problem for studying and planning public policies, due to the inevitable growth in demand for healthcare.^1,2^ Hence, an increase incidence of neurodegenerative diseases can be expected. Neurodegenerative diseases affect specific regions of the nervous system with insidious onset and a relentless progressive course.^3^ They form deposits of proteins that change their conformation, becoming anomalous forms.^4^

This is the case in Alzheimer's disease (AD), Frontotemporal Dementia (FTD), Amyotrophic Lateral Sclerosis (ALS), Parkinson's disease (PD), Dementia with Lewy Bodies (DLB), Multiple System Atrophy (MSA), among others.^4^

AD is the most common neurodegenerative disease and can lead to dementia, significantly compromising memory and other cognitive functions, with sufficient intensity to produce functional loss. Impairments in the execution of daily and social activities, such as recognizing people and places in habitual surroundings are common symptoms.^5-8^ The definitive diagnosis of AD depends strictly on the histopathological examination of the *post-mortem* brain. It is conducted by identifying the selective loss of neurons in specific areas, as well as through the detection of senile plaques (SP) and neurofibrillary tangles (NFT), which involves the presence of proteins such as β-amyloid (Aβ) and hyperphosphorylated TAU, respectively.^5,9^

PD, the second-most-common neurodegenerative disorder, is characterized as a multisystem disease, presenting with motor symptoms: tremor, bradykinesia, changes in balance and muscle tone; and non-motor symptoms: olfactory deterioration, sleep disorders, urinary, gastrointestinal and cardiovascular abnormalities; pain and depression.^10^ Its pathological diagnosis includes selective neuronal loss in the *pars compacta* of the substantia nigra, as well as the presence of Lewy bodies rich in α-synuclein.^11^ Deposits of this protein can also be found in DLB and MSA.^12^

Amongst the different varieties of FTD, three main syndromes may be recognized: behavioral variant frontotemporal dementia (bv-FTD), presenting with personality and behavioral changes; semantic dementia (SD) with fluent language alteration; or progressive nonfluent aphasia (PNFA), characterized by nonfluent language alteration.^13-15^ Despite the impairment-based classification, there is also a clinical, pathological and genetic overlap. For instance, SD cases may develop features of bv-FTD, and there is also an overlap between FTD and other neurodegenerative diseases such as progressive supranuclear palsy (PSP), and corticobasal degeneration (CBD) and ALS.^16^ TAR DNA-binding protein 43 (TDP-43), whose gene is located on chromosome 1, has been linked to the pathogenesis of FTD and ALS. This intranuclear protein is involved in different cellular processes such as gene transcription, alternative splicing, mRNA stability, microRNA biogenesis, cell division and apoptosis. If modified, the molecule changes its pattern of distribution and function throughout the CNS structures.^17^

The Entorhinal Cortex (EC) is the anterior portion of parahippocampal gyrus of the temporal lobe. It is an important connection point between cerebral cortex areas and the hippocampus, converging visual, auditory and somatosensorial information. Conversely, efferent fibers of the hippocampus, originating in the subiculum and CA^1^, return to the EC, participating in the Papez circuit, which relates to emotional reactions. Several neuropathological changes in the EC, such as those occurring in the AD, isolate or disconnect the hippocampus from the rest of cerebral cortex, resulting in severe memory loss.^18^

The aim of this study was to survey neurodegenerative changes detected by abnormal protein deposits in the EC of subjects aged 50 years or older, using Immunohistochemistry reactions (IHC) and to correlate findings with suspected dementia.

## METHODS

**Figure 1:**
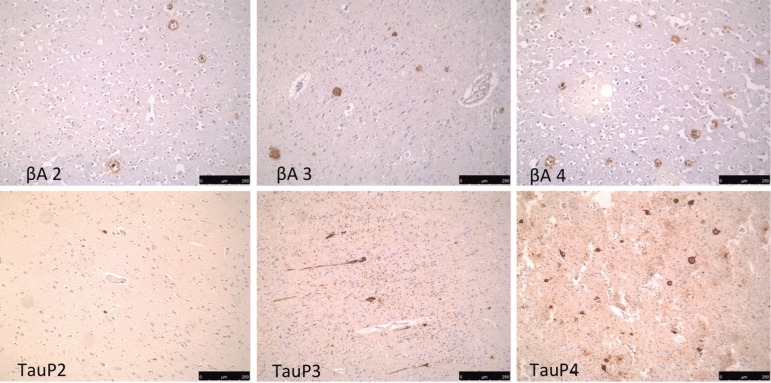
Semi-quantified deposit reactions: βA2: β-amyloid +/sparse neuritic plaques; βA3: β-amyloid ++/moderate neuritic plaques; βA4: β-amyloid +++/frequent neuritic plaques. TauP2: AT^8^ +/sparse neurofibrillary tangles; TauP3: AT^8^ ++/moderate neurofibrillary tangles; TauP4: AT^8^ +++/frequent neurofibrillary tangles.

A descriptive study was conducted in which brain samples were collected at post-mortem autopsies by convenience after informed consent of first-degree relatives. Twelve brains were collected from subjects aged 50 years or older who underwent death verification at the Forensics Department (FD) of Porto Alegre, Southern Brazil. Individuals with violent deaths, subjects whose relatives did not give consent, and those who had acute neurological events (i.e., stroke) as cause of death were excluded. Two brains were extracted from patients followed-up at the Department of Neurology of ISCMPA -- the hospital of the University in Porto Alegre. Donors' clinical information were obtained after death by interviewing the next-of-kin using a questionnaire to evaluate cognitive decline - the Informant Questionnaire on Cognitive Decline in the Elderly (IQCODE) – which has been shown as a reliable screening test for cognitive decline based on neuropathological diagnosis as the criterion.^19^ For the IQCODE, a cut-off point score for dementia of ≥3.27 was adopted, as suggested by Sanchez et al.^20^ All brains were formalin-fixed for 4 weeks and cut using standard protocols. Samples including the EC were embedded in paraffin and 5-µm-thick serial sections cut using a microtome for subsequent use in IHC reactions. Sections were placed on glass slides and deparaffinized in xylene, hydrated using a graded series of ethanol, and immersed in 3% hydrogen peroxide in 100% methanol for 15 min to inhibit endogenous peroxidase activity. To activate the antigens, sections were boiled in 10 mM citrate buffer, pH 6.0, or formic acid. After rinsing in phosphate-buffered saline (PBS), the sections were incubated with normal horse serum for 2 h and then incubated overnight at 4ºC in humid chambers with the primary antibody. The following primary antibodies were used:

(1) anti-Aβ (monoclonal mouse, anti-human beta-amyloid, Dakocytomation, Denmark), dilution 1:25, pretreated for 3 minutes with formic acid;
(2) anti-tau (monoclonal mouse anti-human PHF-tau, at-8, Innogenetics), dilution 1:500, pretreated with citrate for 10 minutes;
(3) anti-α-synuclein (monoclonal mouse anti α-synuclein, Novocastra), dilution 1:200, pretreated with formic acid for 4 minutes and citrate for 20 minutes; and
(4) anti-phospho-TDP-43 (Cosmo Bio Co, Tip-PDT-P05), dilution 1:2500, pretreated with citrate for 20 minutes. After overnight incubation in antibodies, the slides were washed three times in PBS and incubated in DAKO secondary polymer for 40 minutes, Streptavidin HRO, DAKO for 30 minutes, and finally treated for 3 min with 0.01% H_2_O_2_ and 0.05% diaminobenzidine tetrahydrochloride (DAB, Sigma). All slides were counterstained with hematoxylin for 10 seconds, and then evaluated under light microscope for protein deposits by three independent observers. The protein deposit reactions and patterns were semi-quantified using a number scale: 1 (absent), 2 (+), 3 (++) and 4 (+++) (Figure 1). Clinical data was correlated with pathological findings using Fisher's test (significant results p<0.05) or expressed as percentages. The University (UFCSPA) Ethics Committees approved the study.

## RESULTS

**Figure 2:**
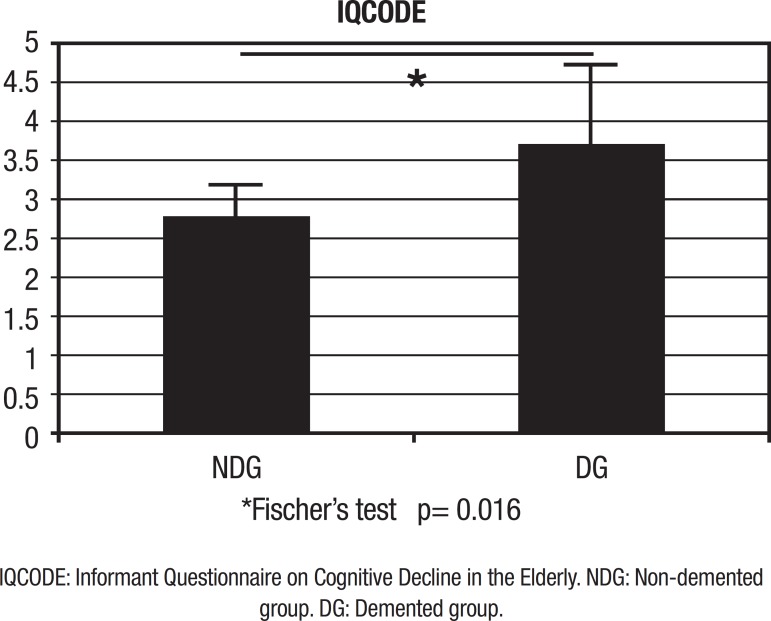
Profile of sample: average IQCODE in groups.

Analyzing the sample of 14 individuals, we found that 57% (8 individuals) exhibited IQCODE results >3.27, compatible with dementia (DG). The mean IQCODE value in the non-demented group (NDG) (6 individuals) was 2.745, while the mean of the DG was 3.72. The DG had a mean age of 72.87 years while the NDG group, 71.16 years. Fifty percent of individuals in each sample (7 individuals) were female (Figure 2).

In the DG, 87.5% (7 individuals) had neuropathological findings that corresponded to ALBP (Alzheimer's-like brain pathology): 14.28% at Braak stages I and II, with CERAD A, 42.8% at stages III and IV, with CERAD A, B or C, and 42.8% at stages V and VI, with CERAD C.^21^ To classify Braak stages, the distribution of NFT was determined using AT^8^ IHC reaction in all brain regions. For CERAD classification, the authors studied neurite plaque density (sparse, moderate or frequent) in the middle frontal gyrus, superior and middle temporal gyri and inferior parietal lobule (data not published).^22^ Twelve and a half percent of subjects (1 individual) from the DG presented a-synucleinopathy, which was an MSA case (diagnosis based on neuropathological findings and clinical manifestations from the medical specialist records kept when patient was in hospital). In addition, 25% (2 individuals) of subjects had phospho-TDP-43-proteinopathy: one case compatible with FTD/ALS (diagnosis based on neuropathological findings and clinical manifestations from the medical specialist records kept when patient was in hospital) associated with ALBP Braak stage I-II, and another case of ALBP Braak stages V-VI. Twenty-five percent (2 individuals) of DG cases showed amyloid angiopathy and 12.5% (1 individual) lacunar stroke.

In the NDG, 16.7% (1 individual) met neuropathological criteria for ALBP Braak stages III-IV. Isolated tauopathy was seen in 66.7% (4 individuals) of the group and isolated β-amyloidopathy in 16.7% (1 individual). The cause of death was also described (Table 1).

**Table 1:** Anatomopathological diagnosis of 14 brains based on the presence and distribution of IHC reaction for β-amyloid, a-synuclein, AT8 (anti-tau) and phospho-TDP-43 in EC.

**Non-Demented Group (NDG)**					
**Cases**	**Sex**	**Age (years)**	IQCODE*	**Anatomopathological diagnosis**	**Cause of death**
020611-1	F	91	1.32	Tauopathy	Pneumonia
020611-2	M	79	3.15	Tauopathy	Cardiac Tamponade
030811	M	56	3.00	Tauopathy	Undetermined
241111	F	89	3.00	ALBP / Braak: III-IV ** CERAD B*** (Tauopathy + β-amyloidopathy)	Multiorgan failure
140312	M	50	3.00	β-amyloidopathy	Undetermined
210612	F	62	3.00	Tauopathy	Undetermined
Average/n(%)*****	F: 50%	71.16 (SD: 17.52)	2.74 (SD: 0.63)	ALBP 16.7%	
**Demented Group (DG)**					
**Cases**	**Sex**	**Age (years)**	IQCODE*	**Anatomopathological diagnosis**	**Cause of death**
120611	F	59	4.52	MSA**** (a-synucleinopathy) and Tauopathy	Septic Shock
050811	F	93	3.33	ALBP / Braak: III-IV ** CERAD A*** (Tauopathy + β-amyloidopathy)	Myocardial Infarction
041111	F	68	3.27	ALBP / Braak: III-IV ** CERAD B*** (Tauopathy + amyloidopathy-β)	Undetermined
160312	M	54	3.86	FTD/ALS*** (TDP-43proteinopathy) ALBP / Braak: I-II ** CERAD A*** (Tauopathy + β-amyloidopathy)	Pneumonia
170312	M	95	4..77	ALBP / Braak: V-VI ** CERAD C*** (Tauopathy + β-amyloidopathy) TDP-43 proteinopathy	Acute pulmonary edema
100412	M	62	3.38	ALBP / Braak: III-IV** CERAD C*** (Tauopathy + β-amyloidopathy) Amyloid angiopathy	Liver cirrhosis
170512	F	81	3.36	ALBP / Braak: V-VI ** CERAD C*** (Tauopathy + β-amyloidopathy)	Undetermined
220712	M	71	3.28	ALBP / Braak: V-VI ** CERAD C*** (Tauopathy + β-amyloidopathy) Amyloid angiopathy Parieto-occipital lacunar stroke	Cardiac tamponade
Average/n(%)	F: 50%	72.87 (SD: 15.37)	3.72 (SD: 0.60)	ALBP: 87.5%	

ALBP: Alzheimer's-like brain pathology (Tauopathy + β-amyloidopathy). SD: Standard deviation.

* IQCODE: Informant Questionnaire on Cognitive Decline in the Elderly;

** Braak classification based on the presence of NFT detected by AT8 (anti-tau) IHC analysis of all brain regions (data not published);

*** CERAD classification based on neuritic plaque density in middle frontal gyrus, superior and middle temporal gyri and inferior parietal lobule (data not published);

**** Diagnosis based on neuropathological findings and clinical manifestations from the medical specialist records kept when patients were in hospital;

***** n(%): frequency (percentage).

**Figure 3:**
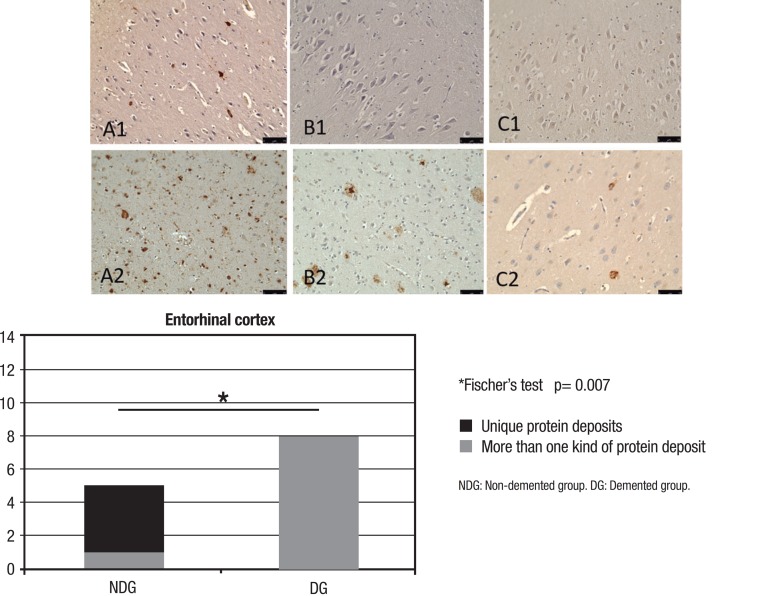
Immunohistochemistry technique showing protein deposits (A: TAU; B: β-amyloid; C: Phospho-TDP-43) in Entorhinal cortex of non-demented (NDG: 1) and demented (DG: 2) groups.

On the semi-quantitative assessment of protein deposits in the EC detected by IHC, only one individual from the whole sample (7.14%) had no protein deposits where this case belonged to the NDG. However, regarding individuals that had deposits in the EC, all individuals from the DG showed deposits of at least two of the proteins studied (Figure 3). The most common association was the presence of both hyperphosphorylated tau and Aβ (87.5% – 7 individuals) proteins, and 25% (2 individuals) of patients had three different reactive proteins (tau, Aβ and phosphoTDP-43). In the NDG group, there were double deposits (hyperphosphorylated tau protein and Aβ) in only one individual (16.7%) (Table 2). There was a statistically significant difference between the presence of unique deposits in the DG and NDG individuals on Fisher's test (significant results p<0.05– Figure 3).

**Table 2:** Positive IHC distribution for Aβ (β-amyloid), TAU (AT8), aSYN (a-synuclein) and Phospho-TDP-43 in EC (Entorhinal cortex) of non-demented (NDG) and demented (DG) groups.

**IHC findings in EC of NDG cases**					
**Cases**	**IQCODE**	**A**β	**TAU**	a**SYN**	**Phospho-TDP-43**
020611-1	1.32	1 (-)	3 (++)	1 (-)	1 (-)
020611-2	3.15	1 (-)	2 (+)	1 (-)	1 (-)
030811	3.00	1 (-)	1 (-)	1 (-)	1 (-)
241111	3.00	2 (+)	2 (+)	1 (-)	1 (-)
140312	3.00	2 (+)	1 (-)	1 (-)	1 (-)
210612	3.00	1 (-)	2 (+)	1 (-)	1 (-)
**IHC findings in EC of DG cases**					
**Cases**	**IQCODE**	**A**β	**TAU**	a**SYN**	**Phospho-TDP-43**
120611	4.52	1 (-)	2 (+)	2 (+)	1 (-)
050811	3.33	2 (+)	4 (+++)	1 (-)	1 (-)
041111	3.27	2 (+)	2 (+)	1 (-)	1 (-)
160312	3.86	2 (+)	2 (+)	1 (-)	2 (+)
170312	4.77	3 (++)	4 (+++)	1 (-)	2 (+)
100412	3.38	2 (+)	2 (+)	1 (-)	1 (-)
170512	3.36	2 (+)	3 (++)	1 (-)	1 (-)
220712	3.28	3 (++)	3 (++)	1 (-)	1 (-)

## DISCUSSION

The DG and NDG showed no statistical differences in relation to gender or age. The predominance of women in the DG is consistent with the fact that the most common dementia (AD) has a higher prevalence in women. It was also assumed that the DG would have a higher mean age in comparison to the NDG, given the incidence of dementia increases with age. However, a larger number of samples can modify this data. The similarity between the two groups allows more reliable comparisons.

The fact that most individuals (57%) had dementia, as determined by the IQCODE, suggests that family members of symptomatic individuals might be more sensitive in agreeing to organ and tissue donation. Close contact with debilitated people, the desire to help, and understanding the need for scientific research to relieve suffering and contribute to a cure in the future are factors positively influencing this decision.^23-25^

As expected, most individuals from the DG (87.5%) presented neuropathological findings of ALBP. In an analysis of the prevalence of dementia in developing countries, 60% of cases were related to AD and approximately 30% of cases to vascular dementia (VD). However, none of these results have been validated by necropsy.^26^ While alive, the diagnosis of probable AD is made by exclusion of other forms of dementia and application of neuropsychological tests, specifically designed to evaluate cognitive ability.^6^ The prevalence of dementia in general, AD in particular, increases significantly with age. A Brazilian epidemiological study in the city of Catanduva (southeast Brazil) evaluated 1,656 people aged over 64 years and diagnosed dementia in 118 patients, corresponding to a prevalence of 7.1%. Dementia was diagnosed in 38.9% individuals in the population over 85 years old. AD was the main cause of dementia, accounting for 55.1% of cases. Although the study has not been validated by necropsy, the prevalence of dementia increased with age and was higher in women, and was inversely correlated with education (3.5% in people with over 8 years' education and 12.2% in illiterate individuals).^6^ However, a study carried out by the Brazilian Aging Brain Study Group (BABSG) of the University of São Paulo found that even a few years of formal education was associated with less cognitive impairment.^27^

Brunnstrom et al. (2009), studying the clinical-pathological concordance in the diagnosis of dementia, showed that 84% of patients with AD symptoms had the significant component of ALBP on the neuropathological examination. This association was slightly weaker for other types of dementia. The overall agreement, considering the mixed dementia findings, between clinical and pathological diagnosis was 63%.^28^ Moreover, neuropathological studies can evaluate the association of several disorders, a common occurrence in the elderly. The most frequently found association was between ALBP and ischemic strokes,^28,29^ seen in one of our cases. There is also evidence that TDP-43 proteinopathy could be related with ALBP, especially in older adults with hippocampal sclerosis (HS).^30-32^ We found this association in two cases without HS, one with ALBP, Braak V-VI classification and the other in a case of FTD with ALS. Glia intracytoplasmic deposits of α-synuclein are typical findings in MAS-Papp-Lantos bodies.^33,34^ We found this proteinopathy associated with NFT (tauopathy) in one case.

In the present study, only one individual, an 89-year-old women who was part of the NDG, had neuropathological findings corresponding to ALBP (Braak stage III-IV, CERAD B), characterizing a preclinical stage or resilience to protein toxicity. Studies show that, on evaluations of non-demented elderly, 45% exhibit neuropathological findings of ALBP.^29,35^

In our sample, only one individual in the NDG, a 56-year-old man, showed no deposits of the proteins studied in the EC. Other patients from the NDG presented at least some protein deposits, mostly single ones (Aβ or hyperphosphorylated tau). Notably, all individuals in the DG showed more than one kind of protein deposit, being ALBP (Aβ and hyperphosphorylated tau) in most cases (87.5% - 7 individuals). AD corresponds to a disease spectrum ranging from asymptomatic lesions in cognitively normal elderly to elderly with classical dementia, in which a transition phase, Mild Cognitive Impairment (MCI), can be identified.^29^ Clinical-pathological correlation studies have been crucial to establish the hypothesis on the pathophysiology of this *continuum* between normal aging and AD. SP primarily occur before the onset of cognitive symptoms, while NFT, neuronal loss and synaptic loss occur in parallel with cognitive decline. However, individuals with asymptomatic AD appear to be resilient to SP and NFT neurotoxicity.^29,36^

The study limitations included the small sample obtained, which was due to the logistical difficulty of collecting brains at the Forensics Department (FD) of Porto Alegre, and the absence of an established donation program that would allow a more extensive interview with family members.^37^ In addition, the fact that no medical information was available for most of the individuals limited the discussion. As strategies to recruit a higher number of donors, we identified the need to provide more detailed information on brain donations, to promote dialogue among families about tissue donation, and to give explanations on how research can contribute to the community and future generations.^38^

The results of this study suggested that deposits of a single type of anomalous protein are normal findings of an aging brain, while more than one kind or the combined presence of anomalous protein deposit indicate the presence of dementia, further measured by the IQCODE.

The systematic comparison of clinical dementia with pathological diagnosis allows essential feedback to improve the diagnostic process and further understanding on the pathophysiology of diseases. The importance of understanding neurodegenerative mechanisms, particularly premature changes in the EC – a vulnerable brain region in cases of dementia – justifies the effort and expense of research, especially in countries such as Brazil.
